# Particle radiotherapy with carbon ion beams

**DOI:** 10.1186/1878-5085-4-9

**Published:** 2013-03-04

**Authors:** Tatsuya Ohno

**Affiliations:** 1 Gunma University Heavy Ion Medical Center, Gunma University, Showa 3-39-22, 371-8511, Maebashi, Gunma, Japan

**Keywords:** Carbon ion radiotherapy, Cancer treatment, High LET, Particle radiotherapy, Personalised medicine

## Abstract

Carbon ion radiotherapy offers superior dose conformity in the treatment of deep-seated malignant tumours compared with conventional X-ray therapy. In addition, carbon ion beams have a higher relative biological effectiveness compared with protons or X-ray beams. The algorithm of treatment planning and beam delivery system is tailored to the individual parameters of the patient. The present article reviews the available literatures for various disease sites including the head and neck, skull base, lung, liver, prostate, bone and soft tissues and pelvic recurrence of rectal cancer as well as physical and biological properties.

## Review

### History

Since the discovery of X-rays by Röntgen in 1895, X-rays, γ-rays and electron beams have been widely used in the management of malignant tumours as a conventional radiotherapy (RT). In 1946, Wilson R. firstly proposed the medical use of proton for cancer therapy, and the first patient was treated at the Lawrence Berkeley National Laboratory (LBNL) in the USA in 1954 [1]. The efficacy of heavy ions for clinical use had been investigated at LBNL between 1977 and 1992, in which most patients were treated with helium and neon ions [1]. In 1994, clinical trial on carbon ion RT (C-ion RT) was launched at the National Institute of Radiological Sciences (NIRS) in Japan. At present, particle beams with protons or carbon ions have been applied gradually in clinics. More than 96,000 patients have been treated with particle beams around the world, of which about 10% were treated with C-ion RT.

### Characteristics of carbon ions

#### Physical aspects

**Figure 1:**
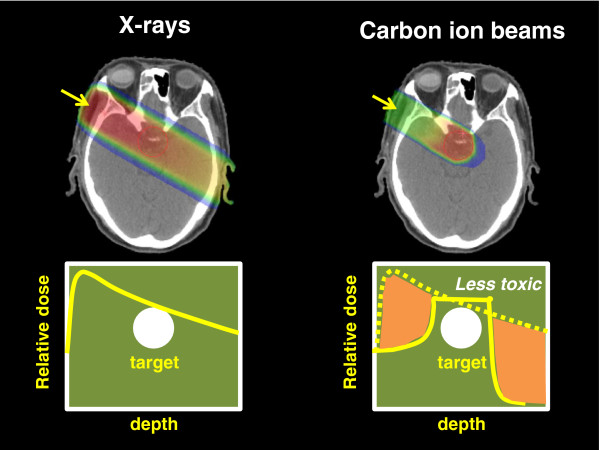
The difference of dose distribution by one port between carbon ion beams and X-rays.

The application of RT is based on the fundamental principle of achieving precise dose localisation in the target lesion while causing minimal damage to surrounding normal tissues. Energy deposition of carbon ion beams increases with penetration depth up to the sharp maximum at the end of their range, known as the Bragg peak. Because the original peak is too narrow and sharp to completely cover the target lesion, broadening of the narrow peak (spread-out Bragg peak (SOBP)) according to the size of the lesion is used in cancer treatment [1,2]. This results in carbon ion beams allowing a highly localised deposition of energy that can be utilised for increasing radiation doses to tumours while minimising irradiation to adjacent normal tissues (personalised cancer treatment). Proton therapy also possesses this property. However, the lateral fall-off around the target is steeper with carbon ion beams than proton beams. In the region beyond the distal end of the peak, almost no dose is deposited with protons, while a small dose is deposited with carbon ions. This is because primary carbon ions undergo nuclear interactions and fragment into particles with a lower atomic number, producing a fragmentation tail beyond the peak [1]. Figure 1 shows the difference of dose distribution by one port between carbon ion beams and X-rays. The algorithm of treatment planning and beam delivery system is tailored to the individual parameters of the patient.

#### Biological aspects

RT works by damaging the DNA of cancer cells. X-rays commonly cause single-strand DNA break, and double-strand DNA break by two hits is essential for cancer cell death. However, cells have mechanisms for repairing single-strand DNA damage, and some of them may survive even after treatment. Carbon ion beams deliver a larger mean energy per unit length (linear energy transfer (LET)) of their trajectory in the body compared with low-LET radiations such as protons or photons. As a result, carbon ion beams, which are high-LET radiation, commonly cause double-strand DNA break by one hit, resulting in the most significant event for cancer cell death [3].

LET has been used to evaluate the biological effects of radiations based on the fact that, as LET increases to 100 keV/μm, the larger relative biological effectiveness (RBE) also increases [4]. LET of neutron beams remains uniform at any depth in the body. In contrast, LET of carbon ion beams increases steadily from the point of incidence in the body with increasing depth to reach a maximum in the peak region. This property becomes a therapeutic advantage when carbon ion beams are used as cancer therapy for deep-seated tumours. In Japan, where a beam-scattering method with a passive beam delivery system is used, RBE of three is assumed at the distal part of the SOBP [5]. In the present review, the dose of carbon ion RT (C-ion RT) is expressed as ‘Gy(RBE)’ (physical carbon ion dose (Gy) × RBE).

High-LET heavy ions also have various biological advantages compared with protons or X-ray beams: decreased oxygen enhancement ratio, diminished capacity for sublethal and potentially lethal damage repairs, reduced cell cycle-dependent radiosensitivity, potential suppression of metastases and efficacy for cancer stem-like cells [6-8]. These characteristics offer theoretical advantage for tumours such as adenocarcinoma, adenoid cystic carcinoma, malignant melanoma and sarcoma that are highly resistant to low-LET irradiation and that sometimes cannot be controlled even with simple dose escalation.

### Clinical results of carbon ion radiotherapy

#### Facilities

At present, there are only five C-ion RT centres in operation in the world [1]: the National Institute of Radiological Sciences in Chiba, Japan, since 1994, Hyogo Ion Beam Medical Center (HIBMC) in Hyogo, Japan, since 2001, the Institute of Modern Physics in Lanzhou, China, since 2006, Heidelberg Ion-Beam Therapy Center (HIT) in Heidelberg, Germany, since 2009 and Gunma University Heavy Ion Medical Center, Gunma, Japan, since 2010 [9]. NIRS is the first C-ion RT facility in Japan using the Heavy Ion Medical Accelerator in Chiba, and it has been used to treat cancers of various sites in more than 6,500 patients. Among the above five facilities, HIBMC and HIT have also performed proton therapy. In addition, at least seven new C-ion RT centres are under development, one in Italy, two in Germany, one in Austria, one in China and two in Japan [1].

#### Head and neck tumour

Head and neck cancer consists of paranasal and sinonasal cancer and cancer of the salivary gland, lip, oral cavity, pharynx and larynx. Histologically, squamous cell carcinoma is the most common histology. However, photon-resistant type of tumours such as adenocarcinoma, adenoid cystic carcinoma, malignant melanoma and sarcoma are sometimes observed. C-ion RT has been employed mainly for locally advanced non-squamous cell carcinomas arising from the paranasal sinus, nasal cavity, salivary gland, pharynx and oral cavity.

Mizoe et al. reported the clinical results of a phase II study of C-ion RT alone in 236 patients with head and neck cancers [10]. Approximately 90% of the patients had locally advanced disease (T3, T4, local recurrence or residual disease after surgery), and they were treated with 57.6 Gy(RBE) in 16 fractions. The 5-year local control rate by histological type was 75% for the 85 patients with malignant melanoma, 73% for the 69 with adenoid cystic carcinoma, 73% for the 27 with adenocarcinoma, 61% for the 13 with papillary adenocarcinoma, 61% for the 12 with squamous cell carcinoma and 24% for the 14 with sarcomas. The 5-year overall survival rate was 68% for adenoid cystic carcinoma, 56% for adenocarcinoma and 35% for malignant melanoma. The 5-year overall survival rate by histological type was 35% for malignant melanoma, 68% for adenoid cystic carcinoma, 56% for adenocarcinoma, 31% for papillary adenocarcinoma, 17% for squamous cell carcinoma and 36% for sarcomas. The 5-year overall survival rate was 68% for adenoid cystic carcinoma, 56% for adenocarcinoma and 35% for malignant melanoma. Although normal tissue reactions included early grade 3 skin and mucosal reactions in approximately 10% of the subjects, late reactions were grade 2 or less. No serious toxicity related to C-ion RT was observed during the follow-up period. This study demonstrated that a relatively higher local control rate was achieved in non-squamous cell carcinomas with acceptable toxicities and that a more intensive approach is required for sarcoma.

Jingu et al. reported the improvement of local control with high-dose (70.4 Gy(RBE) in 16 fractions) C-ion RT for 27 patients with unresectable bone and soft tissue sarcoma of the head and neck [11]. The 3-year local control rate and overall survival rate were 91.8% and 74.1%, respectively. A comparison with historical results showed that the 3-year local control rate with 70.4 Gy(RBE) was significantly higher than that with 57.6 or 64.0 Gy(RBE) (92% vs. 24%, *p* < 0.0001). In addition, the overall survival with 70.4 Gy(RBE) tended to be higher than that with 57.6 or 64.0 Gy(RBE) (74% vs. 43%, *p* = 0.09). Regarding the late toxicities, visual loss was observed in one eye of one patient whose optic nerve was entirely involved by the tumour. Severe pain in the maxillary bone (grade 3) from sequestrum formation was observed in four patients.

Malignant mucosal melanoma in the head and neck has a poor prognosis, the 5-year overall survival rate usually being about 30% or less [12,13], with a low local control rate and frequent distant metastases. At NIRS, based on the poor survival of malignant mucosal melanoma by C-ion RT alone [14], a new protocol of C-ion RT combined with systemic chemotherapy was developed [15]. Forty-six patients with malignant mucosal melanoma in the head and neck were prospectively treated with concurrent C-ion RT (57.6 or 64.0 Gy(RBE) in 16 fractions) and chemotherapy consisting of dacarbazine, nimustine hydrochloride and vincristine (DAV therapy). The 3-year local control rate, distant metastasis-free survival rate and overall survival rate of all patients were 81.1%, 37.6% and 65.3% with a median follow-up time of 19.0 months, showing promising improvement of survival. Further observation will be necessary to confirm the long-term efficacy and toxicities.

#### Skull base tumour

In the treatment of skull base tumours, critical organs such as cranial nerves, eyes, cochlea, brain stem and brain tissue limit the application of high-dose irradiation to the target lesion. Chordomas and chondrosarcomas, known as photon-resistant tumours, have been treated with proton therapy or C-ion RT.

The generally accepted treatment for chordomas of the skull base is resection followed by adjuvant radiation therapy for residual disease. Takahashi et al. recommended a combination of surgical removal of the tumour around the brainstem and the optic nerve with post-operative C-ion RT in order to improve survival and quality of life [16]. Munzenrider et al. reported that the local control rate was 73% at 5 years after proton therapy, and this decreased to 54% at 10 years, indicating the possibility of local recurrence even after 5 years [17]. At NIRS, as a result of a dose escalation study of CIRT for skull base tumours, a dose fractionation of 60.8 Gy(RBE)/16 fractions for 4 weeks was decided as the recommended dose because of acceptable normal tissue reactions and good local tumour control (100%) [18]. The latest data from NIRS demonstrated that the 5-year and 10-year local control rates were 88% and 80% in patients receiving this regimen, and they were without severe grade 3 or more late toxicities [1]. Tsujii pointed out that C-ion RT holds a promising potential of improving long-term results, most likely due to increased biological effects of carbon ions as well as the sharp lateral fall-off permitting better sparing of critical organs [1].

At the Gesellschaft für Schwerionenforschung (GSI) in Darmstadt, Germany, 96 patients with chordoma of the skull base have been treated with C-ion RT [19]. The 5-year local control rate was 70% for the entire population and 100% for 12 patients receiving more than 60 Gy(RBE). The 5-year overall survival rate was 89%. In addition, 54 patients with low-grade and intermediate-grade chondrosarcomas of the skull base have been treated with carbon ion radiation therapy at GSI [20]. Median total dose was 60 Gy(RBE). Only two patients developed local recurrences. The 5-year local control and overall survival rates were 90% and 98%. Therefore, similar excellent local control rates were obtained from both NIRS and GSI experiences.

#### Non-small cell lung cancer

**Figure 2:**
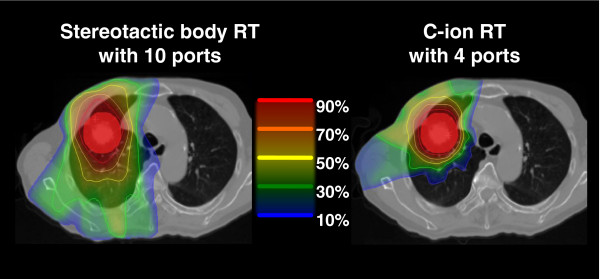
**The difference of composite dose distribution between stereotactic body RT and C-ion RT for stage I non-small cell lung cancer.** The low-dose irradiated volume of lung tissues is lower in C-ion RT than in stereotactic body RT.

Surgery is the standard treatment of choice for early-stage non-small cell lung tumours, but radiotherapy is a good option for those who cannot undergo surgery. For peripheral-type stage I non-small cell lung cancers, the local control rates in T1 (≤3 cm) and T2 (>3 cm) were 64% and 50% for conventional photon therapy [21], 79% to 92% and 30% to 79% for stereotactic body RT [22-24], and 82% and 89% and 49% and 62% for proton therapy [25,26], respectively. Timmerman et al. recently reported that the estimated 3-year primary tumour control and overall survival rates with stereotactic body RT with 54 Gy/three fractions (T1, 80%; T2, 20%) were 98% and 56% [27]. However, grades 3 and 4 toxicities were reported in 13% and 4% of the patients, respectively. Although tumour control is clearly related to radiation dose, higher doses come at the cost of toxicity to normal tissues. Figure 2 shows the comparison of dose distribution between stereotactic body RT and C-ion RT for stage I non-small cell lung cancer. The low-dose irradiated volume of lung tissues is lower in C-ion RT than in stereotactic body RT. In C-ion RT, for peripheral-type stage I non-small cell lung cancers, the fraction number and treatment time have been reduced in gradual steps from 18 fractions/6 weeks through 9 fractions/3 weeks and 4 fractions/1 week and eventually to single-fraction treatment. In 129 patients treated at NIRS with the nine- and four-fraction regimens, there were no serious toxic reactions, their 5-year overall survival rates were 50.0% and 45.0%, and their 5-year local control rates were 95% and 90%, respectively [28,29]. Especially, local control of T2 (tumour size > 3 cm) treated with nine or four fractions was 85%, demonstrating the advantage of C-ion RT for larger tumour control without severe toxicities. Considering the theoretical advantage of C-ion RT, challenging locally advanced non-small cell lung cancer in combination with concurrent chemotherapy is warranted in the future.

#### Hepatocellular carcinoma

In Japan, chronic hepatitis C is associated with 90% of hepatocellular carcinoma (HCC) cases, and HCC is associated with liver cirrhosis in 85% of all cases. The patients with HCC often require repeated therapies due to the multicentric nature of carcinogenesis in liver cirrhosis. Thus, both radical efficacy and minimal invasiveness are required for the treatment of HCC. As local therapy, a variety of treatment modalities such as complete surgical resection, hepatic transplantation, radiofrequency ablation, microwave coagulation therapy, percutaneous ethanol injection and transarterial chemoembolisation have been used so far, but each of them still has its specific limitations especially for larger tumours (>3 cm) [30].

Since the tolerance of the liver to irradiation is generally poor, limited-dose X-ray therapy for HCC has resulted in poor local control and survival. For unresectable HCC patients with or without portal vein tumour thrombosis, the 5-year survival rate ranges from 9% to 25% after 50 Gy [30]. Attempts to improve clinical outcomes for patients with hepatocellular carcinoma have led to the use of charged-particle therapy. Tsukuba University reported that the 3-year local control and survival rates in the porta hepatis group (72.6 Gy(RBE) in 22 fractions) were 86% and 45% [31]. The corresponding rates in the non-porta hepatis group (66 Gy(RBE) in ten fractions) were 95% and 49% [32]. At NIRS, characteristics of the patients undergoing C-ion RT included a disease status for which other therapies appeared to offer no potential of sufficient efficacy or other treatments had proved to be ineffective in local tumour control [33]. In 69 patients treated with 52.8 Gy(RBE) in four fractions for 1 week, post-treatment impairment in hepatic function was minimal. The local control and survival rates at 3 years were 88% and 44% in the porta hepatis group and 96% and 61% in the non-porta hepatis group. Therefore, in comparison with proton therapy, C-ion RT could offer smaller fractionation regimen with comparable high local control.

#### Prostate cancer

Okada et al. reported a retrospective analysis of 740 prostate cancer patients in order to compare late radiation toxicity and biochemical control in different dose fractionation schedules of C-ion RT [34]. C-ion RT was administered at a total dose of 63 Gy(RBE) or 66 Gy(RBE) in 20 fractions for 5 weeks or a dose of 57.6 Gy(RBE) in 16 fractions for 4 weeks. Patients in both the intermediate- and high-risk groups received androgen deprivation therapy (ADT) combined with C-ion RT. Neoadjuvant ADT was administered for 2 to 6 months. Adjuvant ADT was continued for a total duration of 6 months for intermediate-risk patients and for more than 24 to 36 months for high-risk patients. ADT use did not differ by fractionation regimen. Regarding dose fraction, the 5-year biochemical relapse-free (BRF) rate of all patients treated with 16 fractions of C-ion RT (88.5%) was nearly the same as that of those treated with 20 fractions (90.2%). Only one case of late grade 3 genitourinary morbidity was observed in the 20-fraction group, and none of the patients developed late grade 4 or higher complications. The incidence of late grade 2 rectal complications was 3.2% for 66.0 Gy(RBE)/20 fractions, 2.3% for 63.0 Gy(RBE)/20 fractions and 1.5% for 57.6 Gy(RBE)/16 fractions. The incidence of late grade 2 genitourinary complications was 13.6% for 66.0 Gy(RBE)/20 fractions, 6.5% for 63.0 Gy(RBE)/20 fractions and 2.0% for 57.6 Gy(RBE)/16 fractions. Thus, C-ion RT of 57.6 Gy(RBE) in 16 fractions over 4 weeks, which is only half the fractions and time used by most of the intensity-modulated radiation therapies and proton beam therapies, could provide a lower incidence of late rectal toxicity than 20 fractions, with a comparable BRF rate.

#### Bone and soft tissue tumour

A phase I/II dose escalation study was conducted at NIRS in 57 patients with 64 sites of bone and soft tissue sarcomas [35]. At a dose of 73.6 Gy(RBE), 7 of 17 patients developed grade 3 acute skin reaction, and a dose of 70.4 Gy(RBE) or less was recommended for the following phase II study. Although the majority of the tumours were huge (median clinical target volume was 559 cm^3^) and unresectable, local control rates were 88% and 73% at 1 year and 3 years, and overall survival rates were 82% and 46% at 1 year and 3 years. The latest data of 95 patients with medically unresectable sacral chordomas at NIRS showed 5-year local control and overall survival rates of 86% and 88%, respectively [36]. Regarding toxicities, 2 patients experienced severe skin or soft tissue complications requiring skin grafts, and 15 patients experienced severe sciatic nerve complications requiring continuing medication. Based on the analysis of dose-volume histograms, irradiated sciatic nerves of more than 10 cm in length and a total dose of more than 70 Gy(RBE) were possible thresholds for sciatic nerve injury. Considering the fact that the local control rate is about 60% to 80% in total excision cases and 25% to 50% in subtotal resection cases, C-ion RT seems to be a promising alternative to surgery.

The 5-year overall survival rates for patients with osteosarcoma of the trunk who underwent resection were 26% to 41%, while those not receiving surgery were 0% to 10% [37,38]. Matsunobu et al. reported a retrospective analysis of 78 patients with medically inoperable osteosarcoma of the trunk [39]. None of the patients developed grade 3 or 4 late toxicities. The 5-year local control and overall survival rates were 62% and 33%, respectively. Multivariate analysis demonstrated that poor performance status (PS = 2) and large clinical target volume (≥500 cm^3^) were unfavourable prognostic factors for survival. Again, C-ion RT for inoperable osteosarcoma of the trunk could be a promising alternative.

#### Others

C-ion RT has been applied to other cancer sites such as pelvic recurrence after surgery for rectal cancer [40], eye melanoma [41], renal cell carcinoma [42], gynaecological cancers [43,44] and pancreatic cancer [45]. Although most of the cases were photon-resistant tumours, the efficacy of hypofractionated C-ion RT has been demonstrated with acceptable toxicities.

#### Cost-effectiveness

In Japan, where everyone is covered by health insurance schemes, all can receive medical treatment equally. However, medical care costs for malignant neoplasms are escalating. Although C-ion RT is, at least in theory, effective, and promising clinical outcomes based on prospective trials have been reported, due to the high construction and operation costs of the accelerator system, there is still controversy on whether carbon ion RT is too expensive for the potential outcome improvements claimed.

In cooperation with Gunma University Hospital (GUH) and NIRS, the cost-effectiveness of carbon ion radiotherapy was compared with conventional multimodality therapy in the treatment of patients with locally recurrent rectal cancer [46]. Direct costs for diagnosis, recurrent treatment, follow-up, visits, supportive therapy, complications and admission were computed for each individual using a sample of 25 patients presenting with this condition at NIRS and GUH. Patients received only radical surgery for primary rectal adenocarcinoma and had isolated unresectable pelvic recurrence. Fourteen and 11 patients receiving treatment for local recurrence were followed at NIRS and GUH, respectively. Treatment was carried out with C-ion RT alone at NIRS, while multimodality therapy including three-dimensional conformal radiotherapy, chemotherapy and hyperthermia was performed at Gunma University Hospital. The 2-year overall survival rate was 85% and 55% for C-ion RT and multimodality treatment, respectively. The mean cost was 4,803,946 JPY for the C-ion RT group and 4,611,100 JPY for the multimodality treatment group. The incremental cost-effectiveness ratio for C-ion RT was 6,428 JPY per 1% increase in survival, demonstrating the cost-effectiveness of C-ion RT. The median duration of total hospitalisation was 37 days for C-ion RT and 66 days for the multimodality treatment group.

At GSI, the cost-effectiveness of C-ion RT for patients with skull base chordoma was analysed based on the various scenarios for the local control rate and reimbursements of C-ion RT [47]. When local control rate for skull base chordoma achieved with C-ion RT exceeds 70%, the overall treatment costs for C-ion RT are lower than for conventional RT. The cost-effectiveness ratio for C-ion RT is 2,539 euros per 1% increase in survival or 7,692 euros per additional life year.

### Recommendations

Based on the clinical trials conducted at NIRS and GSI, C-ion RT has the following characteristics: (1) By location, C-ion RT is effective in tumours of the head and neck, skull base, lung, liver, prostate, bone and soft tissue sarcoma, etc. (2) By pathological type, it is effective against non-squamous cell types of tumours for which photon therapy has little effectiveness, including adenocarcinoma, adenoid cystic carcinoma, malignant melanoma, sarcoma, etc. (3) Compared with photon therapy, small-fraction regimens (from a single fraction to 16 fractions) can be performed within a short treatment period.

### Outlook

Since the tailor-made treatment planning and beam delivery systems are still developing in the field of C-ion RT, personalised cancer treatment will be further improved in the next decade. Especially, the management of organ motion, tumour shrinkage and deformation and image-guided adaptive treatment strategy will enhance the high precision of beam delivery. Unfortunately, due to the limited number of facilities, most clinical data have been reported from a single institution. In order to increase the impact of evidence level, multi-institutional clinical trials on seeking optimal dose and fractionation of C-ion RT, combined treatments of C-ion RT with existing or developing cancer therapy, and socio-economical impact of small-fraction regimens of C-ion RT will be warranted.

## Conclusions

Based on the unique biophysical characteristics of carbon ion beams, the algorithm of treatment planning and beam delivery system is tailored to the individual parameters of the patient. The biological benefits of C-ion RT have been demonstrated in inoperable cases with various types of sarcoma, adenocarcinoma, adenoid cystic carcinoma and malignant melanoma arising from various sites that are well known as photon-resistant tumours. For non-small cell lung cancer (stage I) and hepatocellular carcinoma, short-course C-ion RT using small fraction resulted in high local control. The potential benefit exists in larger tumour (>3 cm) because low-dose irradiated volume of normal tissues is lower compared with stereotactic body RT and because higher dose with high-LET beams can be given. In intermediate- and high-risk groups of prostate cancer, the regimen with 16 fractions for 4 weeks attained excellent biochemical relapse-free rate without severe late toxicities. On the other hand, definite proportions of the patient population who receive C-ion RT develop distant metastasis even after excellent local control. Further clinical trials consisting of C-ion RT with existing or developing cancer therapy will be required in order to investigate survival benefit.

## Competing interests

The author declares that he has no competing interests.
